# High sampling resolution optical coherence tomography reveals potential concurrent reductions in ganglion cell‐inner plexiform and inner nuclear layer thickness but not in outer retinal thickness in glaucoma

**DOI:** 10.1111/opo.13065

**Published:** 2022-11-23

**Authors:** Janelle Tong, Jack Phu, David Alonso‐Caneiro, Sieu K. Khuu, Michael Kalloniatis

**Affiliations:** ^1^ Centre for Eye Health University of New South Wales New South Wales Sydney Australia; ^2^ School of Optometry and Vision Science University of New South Wales New South Wales Sydney Australia; ^3^ Faculty of Medicine University of Sydney Sydney New South Wales Australia; ^4^ Contact Lens and Visual Optics Laboratory, Centre for Vision and Eye Research, School of Optometry and Vision Science Queensland University of Technology Brisbane Queensland Australia; ^5^ School of Medicine (Optometry) Deakin University Waurn Ponds Victoria Australia

## Abstract

**Purpose:**

To analyse optical coherence tomography (OCT)‐derived inner nuclear layer (INL) and outer retinal complex (ORC) measurements relative to ganglion cell‐inner plexiform layer (GCIPL) measurements in glaucoma.

**Methods:**

Glaucoma participants (*n* = 271) were categorised by 10‐2 visual field defect type. Differences in GCIPL, INL and ORC thickness were calculated between glaucoma and matched healthy eyes (*n* = 548). Hierarchical cluster algorithms were applied to generate topographic patterns of retinal thickness change, with agreement between layers assessed using Cohen's kappa (κ). Differences in GCIPL, INL and ORC thickness within and outside GCIPL regions showing the greatest reductions and Spearman's correlations between layer pairs were compared with 10‐2 mean deviation (MD) and pattern standard deviation (PSD) to determine trends with glaucoma severity.

**Results:**

Glaucoma participants with inferior and superior defects presented with concordant GCIPL and INL defects demonstrating mostly fair‐to‐moderate agreement (κ = 0.145–0.540), which was not observed in eyes with no or ring defects (κ = −0.067–0.230). Correlations (*r*) with MD and PSD were moderate and weak in GCIPL and INL thickness differences, respectively (GCIPL vs. MD *r* = 0.479, GCIPL vs. PSD *r* = −0.583, INL vs. MD *r* = 0.259, INL vs. PSD *r* = −0.187, *p* = <0.0001–0.002), and weak in GCIPL‐INL correlations (MD *r* = 0.175, *p* = 0.004 and PSD *r* = 0.154, *p* = 0.01). No consistent patterns in ORC thickness or correlations were observed.

**Conclusions:**

In glaucoma, concordant reductions in macular INL and GCIPL thickness can be observed, but reductions in ORC thickness appear unlikely. These findings suggest that trans‐synaptic retrograde degeneration may occur in glaucoma and could indicate the usefulness of INL thickness in evaluating glaucomatous damage.

**Key points**

- Concordant changes in macular ganglion cell‐inner plexiform layer thickness and inner retinal layer thickness from optical coherence tomography measurements can be observed across the glaucoma spectrum.
- However, the absence of consistent changes in outer retinal complex thickness indicates that changes in the outer retina as measurable by optical coherence tomography are unlikely.
- The observed inner nuclear layer thickness changes in glaucoma suggest that trans‐synaptic retrograde degeneration may occur in glaucoma and could indicate its usefulness in evaluating glaucomatous damage.

## INTRODUCTION

Glaucoma is a chronic optic neuropathy characterised by progressive damage to the retinal ganglion cell axons.^1^, ^2^ Classically, glaucoma manifests as structural changes in the optic nerve head, retinal nerve fibre layer and retinal ganglion cell bodies, which are clinically observable with funduscopic examination and imaging technologies such as optical coherence tomography (OCT). In particular, the latter has proven popular in clinical and research settings due to its ability to visualise individual retinal layers and determine quantitative reductions in the inner retina, with generally good discrimination between glaucoma and healthy eyes.^3^, ^4^

As glaucoma is chronic and irreversible, there is a prolonged reduction in retinal ganglion cell outputs and associated morphological cell changes, including reduced retinal ganglion cell dendrite density.^5^, ^6^ Reductions in dendritic targets and therefore synaptic connections to amacrine and bipolar cells could theoretically result in associated changes in the upstream outer retina. This phenomenon would be analogous to trans‐synaptic retrograde degeneration observed in the retina, when the primary site of damage is located within the central visual pathways.^7^ Whether this occurs in glaucoma is not well‐established; however, observation of cortical changes signifying anterograde degeneration in glaucoma implies that the reverse, retrograde degeneration, may also be possible.^8^

Whether retinal layers outside of the inner retina are affected in glaucoma is not well‐established, with OCT‐based studies in glaucomatous eyes variably reporting no changes in outer retinal thickness measurements and apparent thickening of outer retinal measurements with increasing glaucoma severity.^9^, ^10^, ^11^, ^12^, ^13^, ^14^, ^15^, ^16^, ^17^, ^18^ The myriad of presentations consistent with glaucoma has likely contributed to widely variable thickness measurements between individual eyes with glaucoma and between cohorts in different studies,^19^, ^20^ and likewise it is well‐documented that retinal thickness measurements in normal eyes are highly variable.^21^, ^22^, ^23^, ^24^, ^25^ Subsequently, when not accounting for variations across individuals, significant overlap in thickness measurement distributions could occur depending on effect size. Following on, previous studies have used data averaged across subfields per the Early Treatment for Diabetic Retinopathy (ETDRS) grid, for example.^9^, ^11^, ^13^ However, the resultant limited spatial resolution has been demonstrated to pool together macular regions displaying different retinal thickness and ageing characteristics in normal eyes,^26^ and similarly may pool together relatively normal and damaged locations, further contributing to apparent variability within glaucoma cohorts.

The present proof‐of‐concept study sought to analyse relationships between the neural retinal layers, namely the ganglion cell‐inner plexiform layer (GCIPL), inner nuclear layer (INL) and outer retinal complex (ORC), consisting of the outer nuclear layer to the retinal pigment epithelium. By extracting retinal thickness data at a high sampling resolution and generating topographic patterns of change in the retinal layers of interest, there is the potential to visualise macular locations demonstrating concurrent glaucomatous damage across retinal layers at levels of detail not possible using previously described methods, which averaged data over relatively large subfields. Investigating retinal changes in glaucoma in detail may enable a better understanding of glaucomatous processes across different stages of the disease spectrum, in turn potentially aiding future technologies to better identify glaucomatous damage.

## METHODS

### Participant characteristics

Participants previously diagnosed with glaucoma (*N* = 271) and healthy participants (*N* = 548) were retrospectively recruited from patients attending the Centre for Eye Health (Sydney, Australia, Table 1). All participants provided written consent for their clinical data to be used for research purposes, as per ethics protocols approved by the University of New South Wales Australia Human Research Ethics Advisory panel. This study adhered to the tenets of the Declaration of Helsinki throughout its duration. Data from all glaucoma participants and 508 healthy participants have been used in part in previous studies.^26^, ^27^, ^28^, ^29^

**TABLE 1:** Demographic characteristics of the glaucoma and healthy cohorts. As 10‐2 visual fields were only performed based on suspected central functional deficits during the clinical examination, 10‐2 visual field data were not available for the healthy cohort.

	*n*	Age (y ± SD, range)	Sex (M:F)	SE (D ± SD)	Eye (OD:OS)	Disc to fovea tilt (° ± SD)	10‐2 MD (dB ± SD)	10‐2 PSD (dB ± SD)	24‐2 MD (dB ± SD)	24‐2 PSD (dB ± SD)
Glaucoma cohort	271	64.44 ± 11.46 (27.00–88.00)	162:109	−0.73 ± 2.10	132:139	6.90 ± 3.60	−2.74 ± 3.69	3.14 ± 3.61	−3.17 ± 4.19	3.90 ± 3.03
Healthy cohort	548	50.36 ± 16.90 (20.00–86.00)	248:300	−0.55 ± 1.94	289:259	6.87 ± 3.36	N/A	N/A	−0.70 ± 2.05	1.82 ± 1.33

Abbreviations: °, degrees; D, dioptres; dB, decibels; F, female; M, male; MD, mean deviation; *n*, number of participants; N/A, not applicable; OD, right eye; OS, left eye; PSD, pattern standard deviation; SD, standard deviation; SE, spherical equivalent; y, years.

Per previously published clinical examination protocols,^30^, ^31^ all patients referred to the Centre for Eye Health undergo comprehensive eye examinations, including slit‐lamp biomicroscopy, dilated funduscopic examination and optic nerve and macular imaging with Cirrus HD‐OCT (Carl Zeiss Meditec, zeiss.com). Other assessments performed on indication, for example in the context of diagnosed or suspected glaucoma, include central corneal thickness measurement (Pachmate, dghtechnology.com), applanation tonometry, gonioscopic examination of the anterior chamber angle and visual field (VF) testing using the 24‐2 Swedish Interactive Threshold Algorithm (SITA) Faster and/or 10‐2 SITA Fast paradigms (Humphrey Field Analyser, zeiss.com).

All participants in the glaucoma cohort had been previously diagnosed with open‐angle glaucoma in at least one eye by a glaucoma specialist ophthalmologist working within the Centre for Eye Health as part of a collaborative care arrangement with optometrists. Per previous studies and modern glaucoma diagnostic paradigms,^30^, ^32^, ^33^, ^34^ the diagnostic criteria applied included (1) funduscopic signs of optic nerve head and peripapillary damage characteristic of glaucoma, including neuroretinal rim thinning and/or notching, cup widening and/or deepening, retinal nerve fibre defects and disc haemorrhage, and (2) patterns of reduction characteristic of glaucoma in retinal nerve fibre layer and/or GCIPL thickness measurements from OCT. Specific criteria related to pretreatment intraocular pressure and the presence of visual field defects corresponding to observed structural damage were not applied, such that normal tension glaucoma and treated preperimetric glaucoma patients, respectively, were included in this study, to enable visualisation of trends across the glaucoma spectrum. All participants had either previously undergone selective laser trabeculoplasty or were using topical ocular hypotensive medication at the time of recruitment. All glaucoma participants also had 10‐2 VFs performed as part of their clinical examination and no evidence of other retinal conditions potentially affecting retinal thickness measurements or segmentation. Meanwhile, healthy participants were identified as those demonstrating no optic nerve or retinal pathology in either eye. Additional inclusion criteria across both cohorts included spherical equivalent refractive error between +6.00 and −6.00 dioptres, no more than −3.00 dioptres of astigmatism and CiOCT signal strength of 7 or greater. Where one eye met the above inclusion criteria, that eye was included for further analyses, while if both eyes met inclusion criteria, one eye was selected at random.

As glaucoma may variably affect the superior, inferior or both hemifields of the VF, optic disc and other structural parameters, the glaucoma cohort were categorised by 10‐2 VF defect type to enable visualisation of corresponding structural change in the studied retinal layers (Table 2). Visual field defects were identified using Hodapp–Parrish–Anderson criteria,^35^ where at least three contiguous points of the pattern deviation probability map were flagged as *p* < 5% with at least one of those points flagged at the *p* < 1% level. Inferior (I) and superior (S) defects were identified as those where a single contiguous VF defective cluster was located in either the inferior or the superior hemifield, respectively, with a maximum 1° encroachment, that is one point, on the opposite hemifield (Figure S1). Ring (R) defects were identified as those with an arcuate pattern of loss in each hemifield, defined as a visible arc‐shaped pattern with greater loss nasally than temporally.^36^ In situations where distinct clusters of VF depression were noted in both hemifields but did not follow an arcuate pattern, these were classified as inferior or superior based on the hemifield containing the larger cluster by number of points. Clear (C) fields indicated those that did not present with statistically significant defects per the above criteria, regardless of mean deviation (MD), which may be reduced despite no clusters of VF defects due to media opacity. Groups were also stratified by mean deviation (MD) in 3 dB intervals.

**TABLE 2:** Demographic characteristics of the glaucoma subcohort stratified by 10‐2 visual field defect type, where mean deviation (MD) labels indicate a MD up to that value.

	*n*	Age (y ± SD, range)	Sex (M:F)	SE (D ± SD)	Eye (OD:OS)	Disc to fovea tilt (° ± SD)	10‐2 MD (dB ± SD)	10‐2 PSD (dB ± SD)
Up to −3.00, C	96	65.29 ± 10.07 (33.00–88.00)	58:38	−0.69 ± 2.18	51:45	6.77 ± 3.17	−0.59 ± 1.09	1.22 ± 0.28
Up to −3.00, I	44	65.70 ± 9.81 (41.00–84.00)	33:11	−0.55 ± 2.13	18:26	6.76 ± 3.98	−1.24 ± 1.18	2.16 ± 1.17
Up to −3.00, S	54	63.06 ± 11.92 (27.00–85.00)	35:19	−0.92 ± 2.04	27:27	7.17 ± 3.37	−1.49 ± 0.99	2.31 ± 1.20
−3.00 up to −6.00, C	3	56.00 ± 5.57 (50.00–61.00)	3:0	−0.83 ± 1.13	1:2	6.22 ± 6.65	−3.68 ± 0.26	1.53 ± 0.21
−3.00 up to −6.00, I	11	64.64 ± 16.92 (32.00–88.00)	6:5	−0.08 ± 2.62	4:7	6.53 ± 2.97	−4.38 ± 0.93	3.58 ± 2.70
−3.00 up to −6.00, S	25	64.20 ± 13.63 (29.00–84.00)	12:13	−0.68 ± 1.99	12:13	6.96 ± 4.53	−4.42 ± 1.09	3.60 ± 3.95
−3.00 up to −6.00, R	5	66.00 ± 15.23 (47.00–85.00)	0:5	−0.55 ± 1.61	1:4	6.75 ± 1.40	−4.31 ± 0.96	4.82 ± 2.21
−6.00 up to −9.00, S	8	61.00 ± 7.56 (49.00–70.00)	4:4	−1.00 ± 2.32	7:1	7.47 ± 5.95	−6.60 ± 0.71	6.98 ± 4.77
−6.00 up to −9.00, R	4	63.75 ± 16.5 (43.00–78.00)	2:2	−1.47 ± 3.20	1:3	9.34 ± 3.11	−7.89 ± 1.20	8.20 ± 4.78
−9.00 or worse, I	2	75.50 ± 0.71 (75.00–76.00)	0:2	0.69 ± 1.15	1:1	8.43 ± 0.61	−12.09 ± 1.17	13.27 ± 0.81
−9.00 or worse, S	12	61.83 ± 17.25 (31.00–81.00)	6:6	−1.20 ± 1.92	7:5	6.13 ± 3.11	−11.67 ± 2.65	12.36 ± 3.63
−9.00 or worse, R	7	64.29 ± 8.71 (48.00–73.00)	3:4	−1.11 ± 1.90	2:5	7.18 ± 5.20	−15.00 ± 4.88	11.98 ± 1.37

Abbreviations: °, degrees; C, clear; D, dioptres; dB, decibels; F, female; I, inferior; M, male; MD, mean deviation; *n*, number of participants; OD, right eye; OS, left eye; PSD, pattern standard deviation; R, ring; S, superior; SD, standard deviation; SE, spherical equivalent; y, years.

### OCT segmentation and thickness map generation

For participants in both the glaucoma and the healthy cohorts, macular cube scans acquired using the Cirrus HD‐OCT during the clinical examination were exported in image format from the included eye. Macular cube scans span 6.00 mm × 6.00 mm, with the corresponding resolution spanning 512 pixels wide and 128 pixels high, from 128 horizontal B‐scans. Corresponding extensible markup language (XML) files were exported for each scan, containing additional information such as the location of the foveal centre and laterality.

**FIGURE 1:**
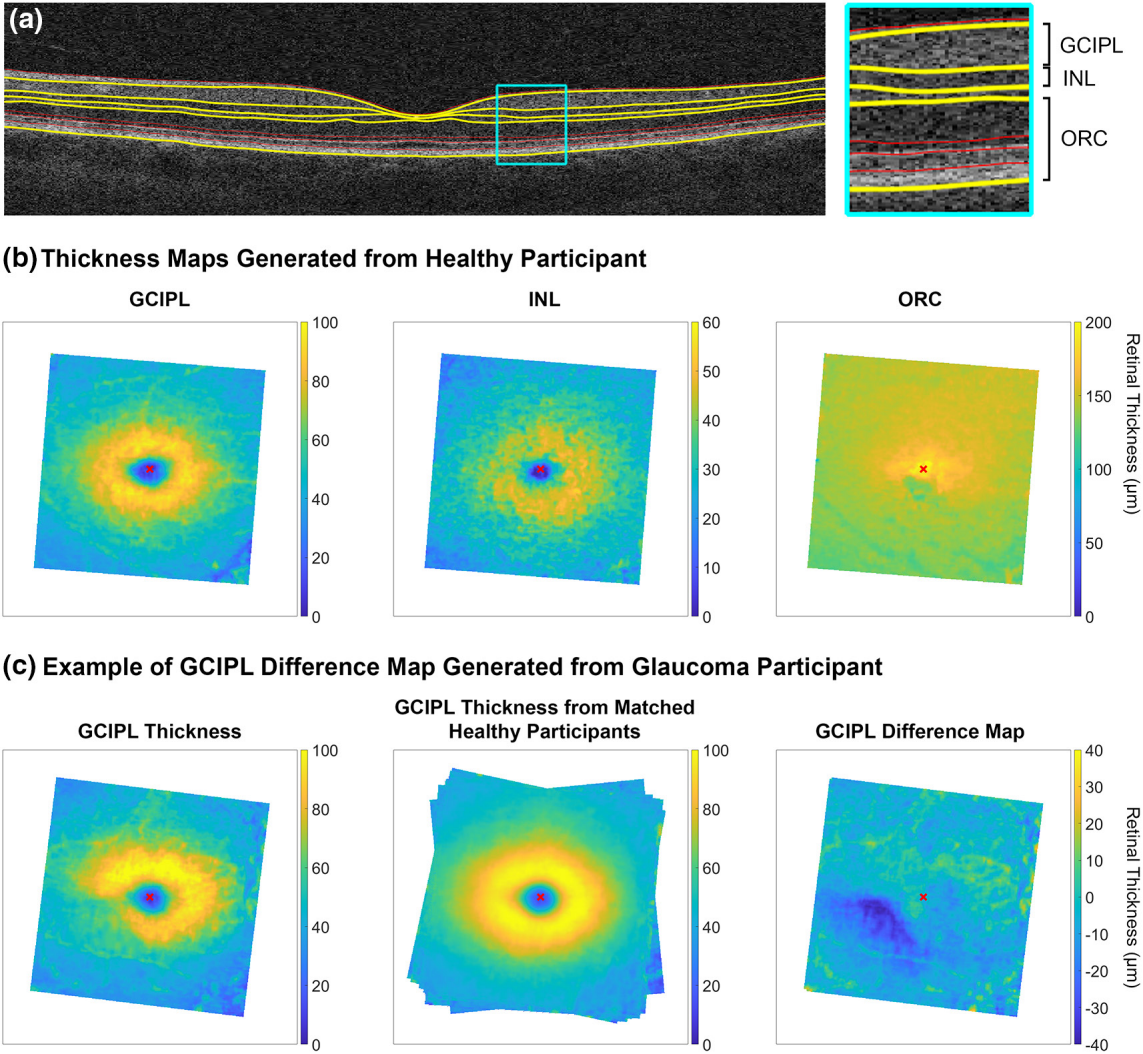
Methods used to generate deviation data, or difference maps of retinal thickness, for the glaucoma cohort. Note the different scales for each map, despite similar colours used in each for simplicity. (a) Segmentation of nine retinal boundaries per the ocular coherence tomography (OCT) segmentation boundaries in an example healthy participant, with those delineating the ganglion cell‐inner plexiform layer (GCIPL), inner nuclear layer (INL) and outer retinal complex (ORC) highlighted in yellow. (b) GCIPL, INL and ORC thickness maps generated from layer segmentations for the same participant in a., with foveal centres marked with red crosses. Maps were rotated such that the optic disc to fovea tilt was 0°, to enable interindividual structural comparisons. (c) Process used to generate deviation data or difference maps for an example glaucoma participant. GCIPL thickness maps were generated for both the glaucoma participant and from demographics‐matched healthy eyes, based on the outcomes of multiple linear regression analyses, with the variable angles at the peripheral edges of the matched healthy eyes map indicating different rotations due to the various optic disc to fovea tilts between participants. The difference between these two maps was taken to generate the final difference map, with these data used in subsequent analyses.

Segmentation of retinal boundaries was performed using OCT Segmentation Version 2.11, an open‐source software tool available as part of the Automated Retinal Analysis (AURA) tool (nitrc.org/projects/aura_tools/; provided in the public domain by Neuroimaging Tools and Resources Collaboration, University of Massachusetts Medical School, umassmed.edu).^37^ Pixel‐wise GCIPL, INL and ORC thickness measurements at a resolution of 512 × 128 pixels were subsequently extracted from between the corresponding segmented boundaries using an algorithm written with MATLAB Version R2021b (mathworks.com; Figure 1). As the OCT Segmentation tool does not allow for manual correction of segmentation errors, in the presence of visible segmentation error due to poor signal strength or anatomical artefacts, such as blood vessel shadowing, the corresponding retinal thickness measurement was excluded from further analyses.

Data were resized to 512 × 512 pixel resolution using bicubic interpolation, compensating for differences in meridional scan resolution in the raw macular cube data. Subsequently, to limit anatomical variability and facilitate interindividual comparisons, data were converted to right eye format and rotated around the foveal centre such that the disc to fovea tilt was 0°.^22^, ^38^ Due to lateral displacement of bipolar and retinal ganglion cells from connecting photoreceptors at the central macula, correction of ORC position to coincide with the locations of the connecting INL and GCIPL components was performed using eccentricity‐dependent data described in Figure 3 from Drasdo et al.^39^ While Figure 6 from this paper, describing the computed displacement of retinal ganglion cells averaged across meridians, has been commonly applied in previous studies,^27^, ^33^, ^40^, ^41^ meridian‐specific differences in displacement may be required for more accurate topographical comparisons with functional data.^42^, ^43^

### Demographic features influencing interindividual retinal thickness variations

To determine sources of normal interindividual variation in the investigated retinal layers, mean GCIPL, INL and ORC thickness measurements across the entire scan area were calculated for each participant in the healthy cohort. Given age, sex and refractive error have been variably reported as influencing retinal thickness measurements,^22^, ^23^, ^26^, ^28^, ^44^, ^45^, ^46^, ^47^ multiple linear regression analyses with mean GCIPL, INL and ORC were performed using Graphpad Prism Version 9.1.2 (graphpad.com), with these variables set as main effects and retinal thickness as dependent variables. Backwards stepwise elimination was then performed to ensure multiple linear regression analyses did not inflate the contribution of each demographic variable on retinal thickness measurements. This process involved repeating multiple linear regression with removal of the least significant main effect, with a 10% minimum difference in coefficients pre‐ and post‐removal indicating that the removed main effect contributed sufficiently to be included in the final model.^48^, ^49^

### Retinal thickness deviations and cluster analysis in the glaucoma cohort

For each participant in the glaucoma cohort, quantitative, location‐specific differences in GCIPL, INL and ORC thicknesses relative to a matched subgroup of the healthy cohort were calculated across the entire scan area. Deriving deviations in retinal thickness from a demographic‐matched healthy subgroup was performed to reduce interindividual variability, opposed to adjustment of absolute retinal thickness measurements using coefficients derived from multiple linear regression analyses, as non‐linear models have been previously applied to describe age‐related changes in the GCL, INL and ORC.^22^, ^26^, ^28^, ^50^ These models suggest accelerated decline in retinal thickness with increasing age, and therefore applying linear correction factors may overestimate retinal thickness measurements in older participants. Moreover, raw deviations in retinal thickness were used in subsequent analyses, as deviations normalised as a percentage of healthy eye data would exaggerate small differences in retinal thickness adjacent to the foveal pit and at the peripheral macula, where absolute measurements approach the measurement floor.^51^ For each retinal layer, the selection of a matched subgroup from the healthy cohort was dependent on the outcomes of the multiple linear regression analysis for that retinal layer; where age and refractive error were identified as significant, for each glaucoma participant the matched subgroup consisted of healthy participants aged within ±7.5 years and with refractive error within ±2.00 dioptres of that participant. Then, for each pixel location in the entire scan area, the mean retinal thickness measurement was calculated over all scans from the matched subgroup as the normative comparison measurement, and the deviation in retinal thickness at this location for the glaucoma participant's scan was computed.

Across glaucoma subcategories, cluster analysis methods were applied to the retinal thickness deviation maps to identify locations demonstrating statistically similar reductions in retinal thickness; in turn enabling visualisation of trends in reductions across the studied retinal layers. From 512 × 512 pixel resolution, deviation data were averaged across grid squares measuring 8 × 8 pixels in size, equivalent to 93.75 × 93.75 μm, for a total grid measurement area of 64 × 64 squares. This was conducted to minimise risk of aliasing with cluster analysis without compromising on sampling resolution, and Khou et al.^52^ have previously demonstrated that a similar sampling resolution produced eccentricity‐dependent patterns similar to those from human histological data.^53^, ^54^ For each glaucoma subcohort, hierarchical cluster analysis was performed using SPSS Statistics Version 23.0 (ibm.com). This approach groups data points into discrete clusters based on similarity in numerical properties and has previously been used to identify spatial locations with similar ageing characteristics in the visual field, macular thicknesses derived from OCT and corneal parameters.^26^, ^28^, ^55^, ^56^ Initially, each data point falls within individual clusters, which are then consecutively merged in the order of most‐to‐least similar in a systematic manner until the endpoint, where all data points fall into a single cluster. The similarity between clusters determining order of cluster merging was calculated using within‐clusters linkage and squared Euclidean distance.

A key advantage of hierarchical cluster analysis, over other cluster methods such as K‐means, is that no *a priori* assumptions are made on the most suitable number of clusters due to its agglomerative technique. Rather, predefined statistical criteria are applied to determine the optimal number of clusters from hierarchical cluster results, and in this study, two distinct criteria were implemented. First, to avoid excessive complexity and therefore overassignment due to the volume of data input to the clustering algorithm, the Bayesian information criterion based on the likelihood function was applied as a starting point for the maximum number of suitable clusters.^50^, ^57^ Data were then classified according to this number of clusters from hierarchical cluster analysis results, and d’ was calculated for each cluster pair, based on the mean (*x*) and standard deviation (*σ*) for each cluster^28^, ^55^:

$$d^{\prime }=\frac{|x_{1}-x_{2}|}{\sqrt{0.5\times \left(\sigma_{1}^{2}+\sigma_{2}^{2}\right)}}$$

The d′ criterion was set at 1, indicating at least 1 standard deviation separating cluster means; cluster pairs were consecutively merged should the d′ for a cluster pair be <1, until all cluster pair comparisons met this criterion.

### Statistical analysis

Statistical analyses were performed using Graphpad Prism, SPSS Statistics Version 23.0 and MATLAB Statistics and Machine Learning Toolbox (mathworks.com). To facilitate quantitative comparisons between cluster patterns generated from the GCIPL, INL and ORC, for each VF defect category, clusters were classified as defective when the cluster mean was negative, indicating a reduction from the matched normative data, and fell below the averaged retinal thickness deviation across the entire macula (Table S1). Clusters not meeting both criteria were classified as not defective. Individual macular locations across the 64 × 64 grid were then classified according to their corresponding cluster assignment, and percentage agreement and Cohen's kappa (κ) were calculated for GCIPL‐INL, GCIPL‐ORC and INL‐ORC combinations across the entire macula for each visual field defect category.

To determine whether quantitative deviations in INL and ORC thicknesses corresponded to regions of GCIPL reduction in the glaucoma cohort, locations were pooled by defective versus not defective GCIPL locations, per criteria as described above. For each glaucoma participant and for the GCIPL, INL and ORC, the mean difference between data within and outside defective GCIPL clusters was calculated. To evaluate whether differences within and outside defective GCIPL clusters for each retinal layer changed as a function of glaucoma severity, Spearman's correlations were applied between mean differences and 10‐2 MD and pattern standard deviation (PSD), which were performed over Pearson's correlations due to non‐normative distributions of MD and PSD values. Both MD and PSD were used in analyses of glaucoma severity due to the clinical utility of each parameter. Specifically, PSD is useful for the identification of early glaucomatous VF results especially when MD is relatively preserved, suggested by its inclusion in staging criteria for early glaucoma.^58^ However, the caveat with performing correlation analyses using PSD is that its magnitude does not increase monotonically with disease severity, instead decreasing once MD exceeds −20 dB with increasing uniformity of the depth of the VF defect.^59^, ^60^ As a result, PSD may underestimate VF severity in advanced disease.^61^

To investigate the relationships between GCIPL, INL and ORC at a more location‐specific level, for each participant, Spearman's correlations between GCIPL and INL, GCIPL and ORC, and INL and ORC were derived across the total 64 × 64 grid measurement area, rather than pooled over clusters. Across the entire glaucoma cohort, the resultant *r* values, indicating the strength of the correlation between each retinal layer comparison, were then plotted against 10‐2 MD and PSD, with further Spearman's correlations applied to these relationships to similarly evaluate changes with increasing glaucoma severity. The level of statistical significance was set as *p* < 0.05 throughout the study.

## RESULTS

### Factors influencing normal variations in retinal thickness

Demographic characteristics influencing GCIPL, INL and ORC thickness were investigated in the healthy cohort, to limit sources of normal interindividual variability within the glaucoma cohort and enhance identification of trends. Multiple linear regression analyses revealed that parameters for age, refractive error and sex were significant across all retinal layers; however, with the elimination of sex, there was no appreciable difference in the age and refractive error parameters in the GCIPL and INL, indicating that sex did not significantly contribute to the original regression model (Table 3). By contrast, with the elimination of refractive error in analyses of the ORC, differences in parameter estimates for age and sex were 20.12% and 30.23%, respectively. These analyses indicated that when generating deviation maps between the glaucoma cohort and matched subcohorts from healthy eyes, matching for age and refractive error was required for the GCIPL and INL, while matching for age, refractive error and sex was required for the ORC.

**TABLE 3:** Comparison of multiple linear regression coefficients without and with backwards stepwise elimination, that is elimination of the least significant variable denoted by N/A (not applicable)

	GCIPL		INL		ORC	
Multiple linear regression	Parameter estimate ± SE	*p* Value	Parameter estimate ± SE	*p* Value	Parameter estimate ± SE	*p* Value
Age	−0.114 ± 0.012	<0.0001	−0.040 ± 0.004	<0.0001	−0.098 ± 0.017	<0.0001
Rx	−0.914 ± 0.375	0.02	0.248 ± 0.033	<0.0001	0.437 ± 0.146	0.003
Sex	0.494 ± 0.104	<0.0001	−0.476 ± 0.120	<0.0001	−1.821 ± 0.525	0.0006
**With backward stepwise elimination**	**Parameter estimate ± SE**	**Difference (%)**	**Parameter estimate ± SE**	**Difference (%)**	**Parameter estimate ± SE**	**Difference (%)**
Age	−0.112 ± 0.012	1.67	−0.039 ± 0.004	2.46	−0.078 ± 0.016	20.12
Rx	0.513 ± 0.104	−3.83	0.258 ± 0.034	−3.99	N/A	N/A
Sex	N/A	N/A	N/A	N/A	−1.953 ± 0.526	30.23

*Note*: Differences between corresponding coefficients exceeding 10% indicated that the eliminated variable contributed meaningfully to the multiple linear regression equation, and therefore to normative variation in that retinal layer.

Abbreviations: %, percentage; SE, standard error; Rx, refractive error.

Derivation of healthy subgroups from the entire normative database of 548 participants, matched to each individual glaucoma participant based on the above demographic factors, produced a minimum of three matched healthy participants per glaucoma participant (Figure S2). Due to increasing prevalence of ocular pathology with increasing age, older glaucoma participants tended to have fewer participants in their corresponding healthy subgroup, with a greater difference in glaucoma participant age and mean subgroup age. However, based on the maximum difference between glaucoma participant and mean subgroup age, the maximum predicted difference in retinal thicknesses due to age was 3.13% in the GCIPL, −2.26% in the INL and −0.54% in the ORC only (−1.87 μm, −0.65 μm and −0.68 μm, respectively, Supplementary Analyses S1). Overall, this suggests that potential age differences are likely to contribute little to differences in retinal thickness between glaucoma participants and their matched healthy subgroups.

### Cluster analysis of glaucoma deviation maps

**FIGURE 2:**
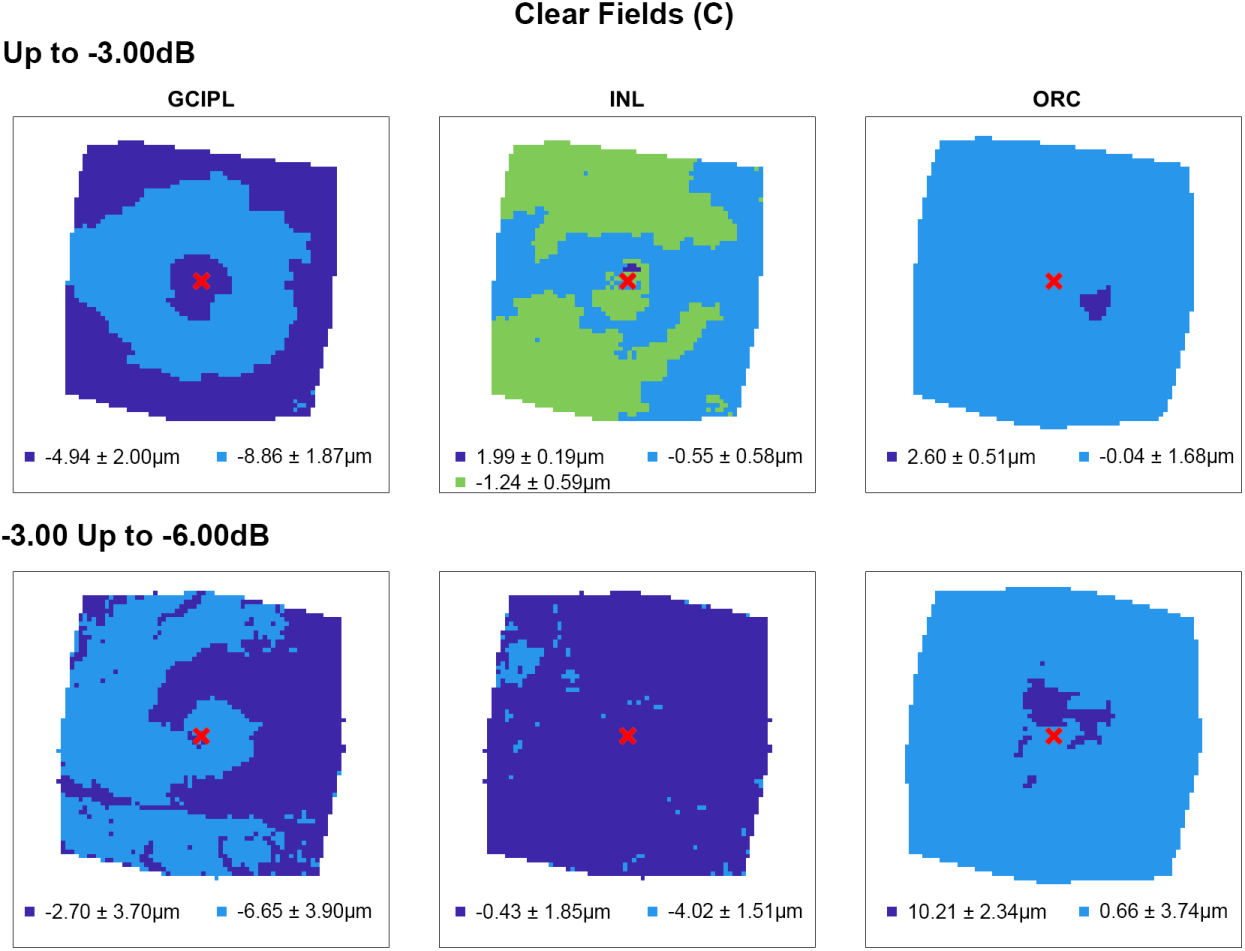
Cluster patterns generated from difference maps in the glaucoma subcohort displaying no visual field defects (clear fields, C), stratified by mean deviation criteria (rows). Locations coloured the same indicate those that showed no significant difference in retinal thickness deviations from the healthy cohort, matched for demographic variables as identified by multiple linear regression analyses (Table 3). While colours representing each cluster are repeated across panels, these do not reflect the same classification across conditions but a nominal classification for each panel and were applied in an ordinal manner for consistency across panels (with the dark blue representing the most positive mean thickness, the light blue the next most positive, etc.). Mean ± SD retinal thickness deviations in μm are shown for each cluster.

**FIGURE 3:**
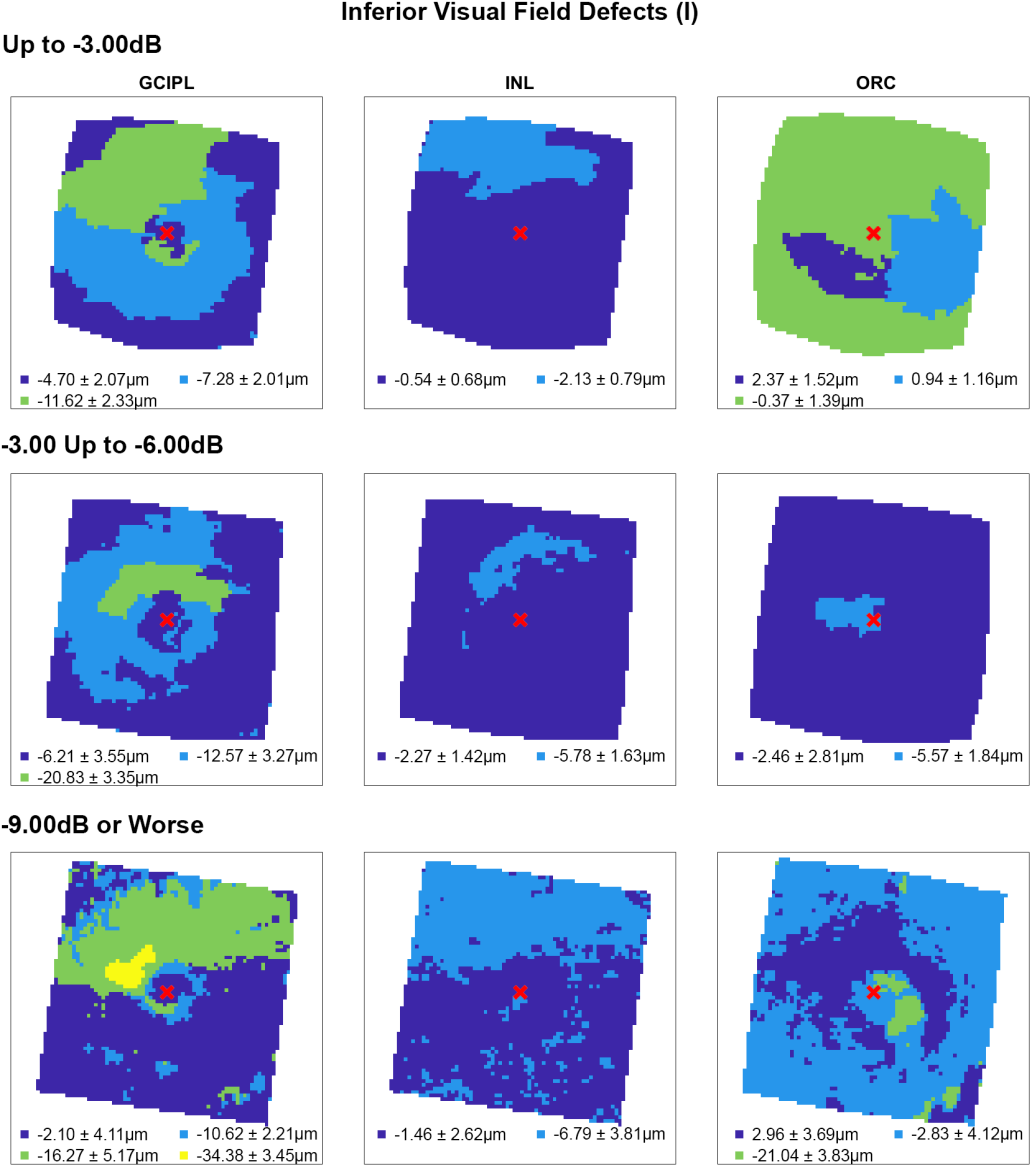
Cluster patterns generated from difference maps in the glaucoma subcohort displaying inferior visual field defects (I), stratified by mean deviation criteria (rows). Per Figure 2, locations coloured the same indicate those that showed no significant difference in retinal thickness deviations from the healthy cohort, matched for demographic variables as identified by multiple linear regression analyses (Table 3). Mean ± SD retinal thickness deviations in μm are shown for each cluster.

**FIGURE 4:**
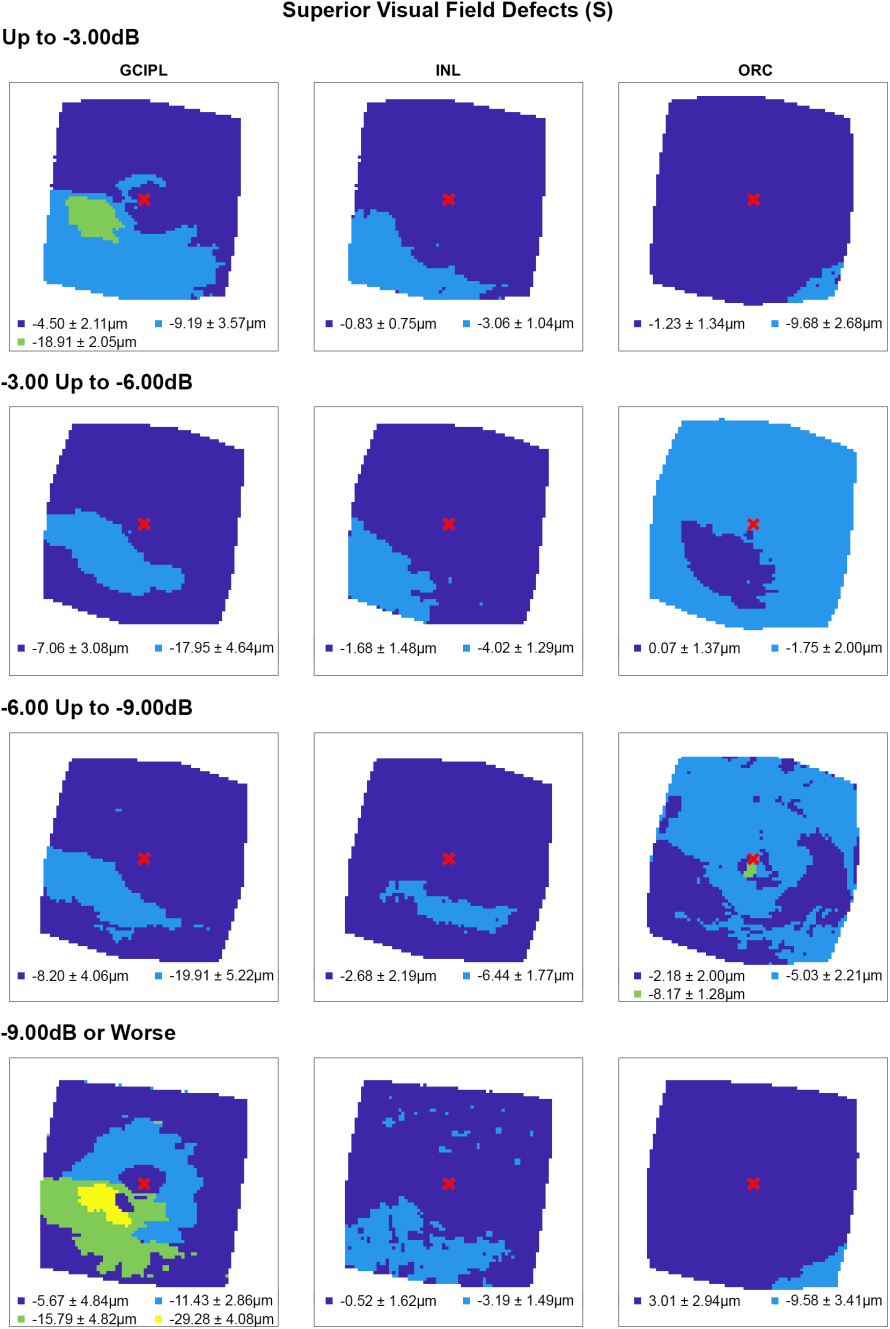
Cluster patterns generated from difference maps in the glaucoma subcohort displaying superior visual field defects (S), stratified by mean deviation criteria (rows). Per Figure 2, locations coloured the same indicate those that showed no significant difference in retinal thickness deviations from the healthy cohort, matched for demographic variables as identified by multiple linear regression analyses (Table 3). Mean ± SD retinal thickness deviations in μm across each cluster

**FIGURE 5:**
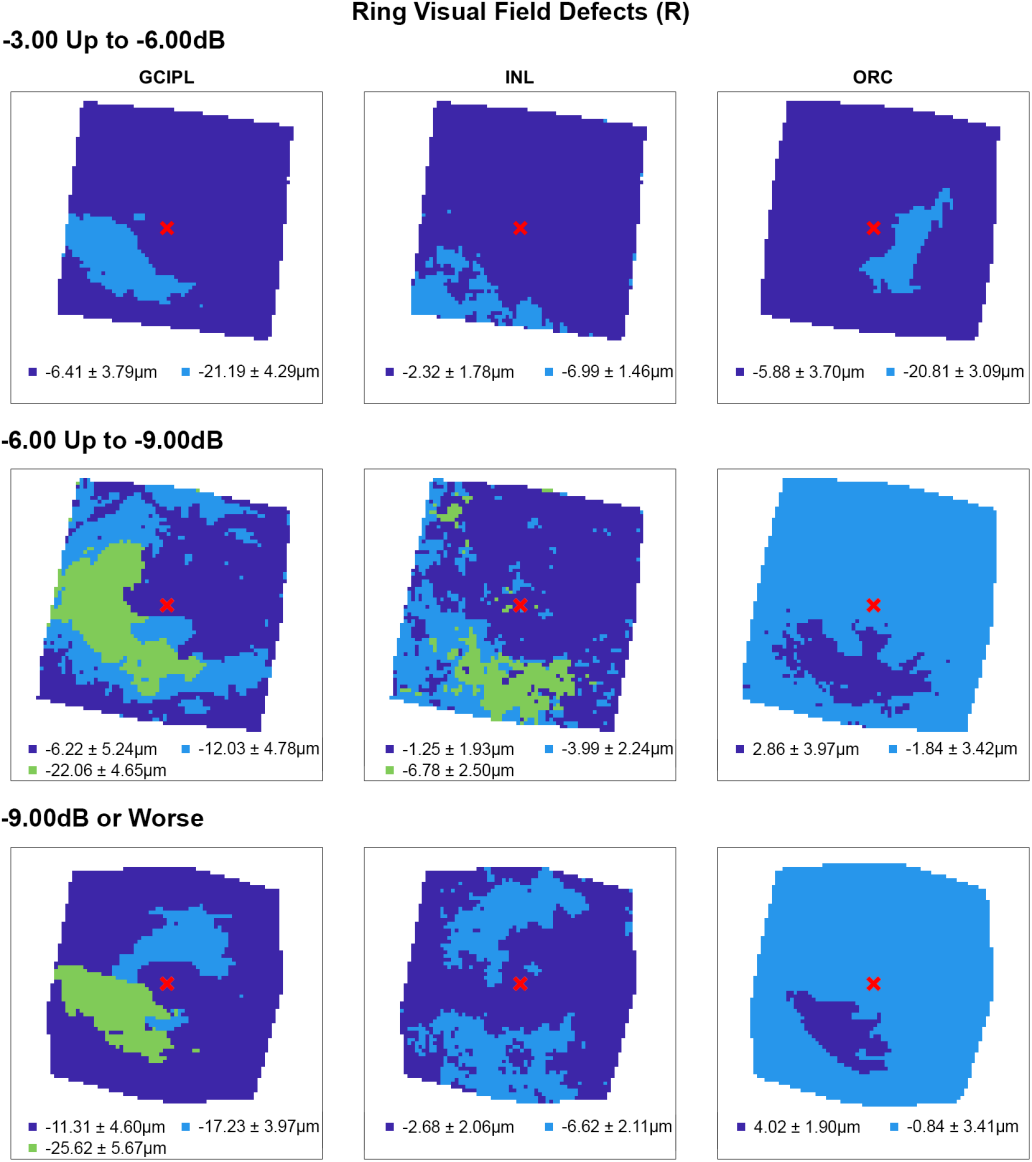
Cluster patterns generated from difference maps in the glaucoma subcohort displaying visual field defects affecting both hemifields (ring defects, R), stratified by mean deviation criteria (rows). Per Figure 2, locations coloured the same indicate those that showed no significant difference in retinal thickness deviations from the healthy cohort, matched for demographic variables as identified by multiple linear regression analyses (Table 3). Mean ± SD retinal thickness deviations in μm are shown for each cluster.

Following multiple linear regression analyses, hierarchical cluster algorithms were applied to GCIPL, INL and ORC deviation maps from glaucoma participants categorised by 10‐2 defect type, to visualise topographic patterns of retinal thickness changes in the macula. Across all retinal layers and glaucoma subcohorts, at least two statistically separable clusters were identified per d’ and Bayesian information criteria (Figures 2, 3, 4, 5 and Table S1). In glaucoma participants with no VF defects per Hodapp–Parrish–Anderson criteria on 10‐2, across MD categories, ring‐like patterns of change were noted in GCIPL deviation maps with no visible corresponding patterns in INL and ORC deviation maps, with relatively poor agreement across GCIPL‐INL and GCIPL‐ORC comparisons (percentage agreement 45.4%–47.9%, κ = −0.038 to 0.03, Table 4). These findings are likely reflective of the relatively early stage of disease and/or heterogeneity in locations of structural macular damage corresponding to clear VF results. Similarly, in participants with ring (both superior and inferior) 10‐2 VF defects, inferior or combined superior and inferior arcuate reductions were noted in the GCIPL deviation map with variable concordance to INL and ORC deviation maps. While in participants with MD up to −6.00 dB percentage agreement appeared high between GCIPL‐INL and GCIPL‐ORC comparisons (81.0%–82.3%), this is likely related to the relatively large area classified as not defective, and Cohen's κ values indicated no‐to‐fair agreement only across all MD categories (κ = −0.067 to 0.23).^62^ These findings likely reflect variable superior and inferior macular involvement in these participants. By contrast, glaucoma participants with inferior or superior VF defects, generally presented with superior or inferior arcuate GCIPL defects, respectively, with wider and/or deeper defects noted with worsening MD. Interestingly, in these participants, clusters of reduced INL thickness were observed in locations generally corresponding to clusters of reduced GCIPL thickness, albeit these clusters appeared narrower than those in the GCIPL. Percentage agreement in GCIPL‐INL comparisons was also generally higher than other comparisons (62.9%–87.0%), while Cohen's κ values were fair to moderate across MD categories (κ = 0.389–0.540). By contrast, in cluster locations with greater GCIPL and INL reduction, either no corresponding cluster in the ORC was observed, indicating no consistent change in ORC deviation across participants in that subcohort, or colocalised clusters demonstrated thicker ORC relative to other macular locations. These were reflected in variable percentage agreements across comparisons and Cohen's κ values indicating no to fairly negative agreement (κ = −0.236 to 0.174), with the exception of participants with MD up to −3.00 dB and inferior defects where fair agreement was observed (κ = 0.222).

**TABLE 4:** Agreement parameters for comparisons of cluster patterns across different retinal layer pairs, across all visual field defect categories

	GCIPL‐INL comparisons		GCIPL‐ORC comparisons		INL‐ORC comparisons	
	Percentage agreement (%)	Cohen's κ, *p* value	Percentage agreement (%)	Cohen's κ, *p* value	Percentage agreement (%)	Cohen's κ, *p* value
Up to −3.00, C	47.9	−0.038, 0.024	46.0	−0.021, <0.0001	55.1	0.024, <0.0001
Up to −3.00, I	79.3	0.432, <0.0001	53.0	0.222, <0.0001	47.5	0.174, <0.0001
Up to −3.00, S	78.1	0.495, <0.0001	59.8	−0.001, 0.847	79.9	−0.036, <0.0001
−3.00 up to −6.00, C	47.3	0.030, <0.0001	45.4	N/A	96.5	N/A
−3.00 up to −6.00, I	62.9	0.145, <0.0001	57.9	0.020, 0.005	89.6	−0.035, 0.024
−3.00 up to −6.00, S	78.2	0.189, <0.0001	8.4	−0.248, <0.0001	21.1	−0.05, <0.0001
−3.00 up to −6.00, R	82.3	0.123, <0.0001	81.0	−0.044, 0.006	82.3	−0.095, <0.0001
−6.00 up to −9.00, S	87.0	0.389, <0.0001	32.3	−0.151, <0.0001	36.9	−0.034, <0.0001
−6.00 up to −9.00, R	61.9	0.230, <0.0001	36.9	−0.236, <0.0001	25.2	−0.212, <0.0001
−9.00 or worse, I	77.7	0.540, <0.0001	38.8	−0.163, <0.0001	45.5	−0.016, 0.22
−9.00 or worse, S	79.0	0.357, <0.0001	75.6	−0.059, <0.0001	76.9	−0.051, <0.0001
−9.00 or worse, R	56.5	−0.067, <0.0001	19.2	−0.131, <0.0001	32.1	−0.052, <0.0001

*Note*: Not applicable (N/a) indicates Cohen's kappa (κ) analyses that could not be performed as all ORC locations were classified as not defective.

Abbreviations: %, percentage; C, clear; GCIPL, ganglion cell‐inner plexiform layer; I, inferior; INL, inner nuclear layer; ORC, outer retinal complex; R, ring; S, superior.

### Relationships between GCIPL, INL and ORC

**FIGURE 6:**
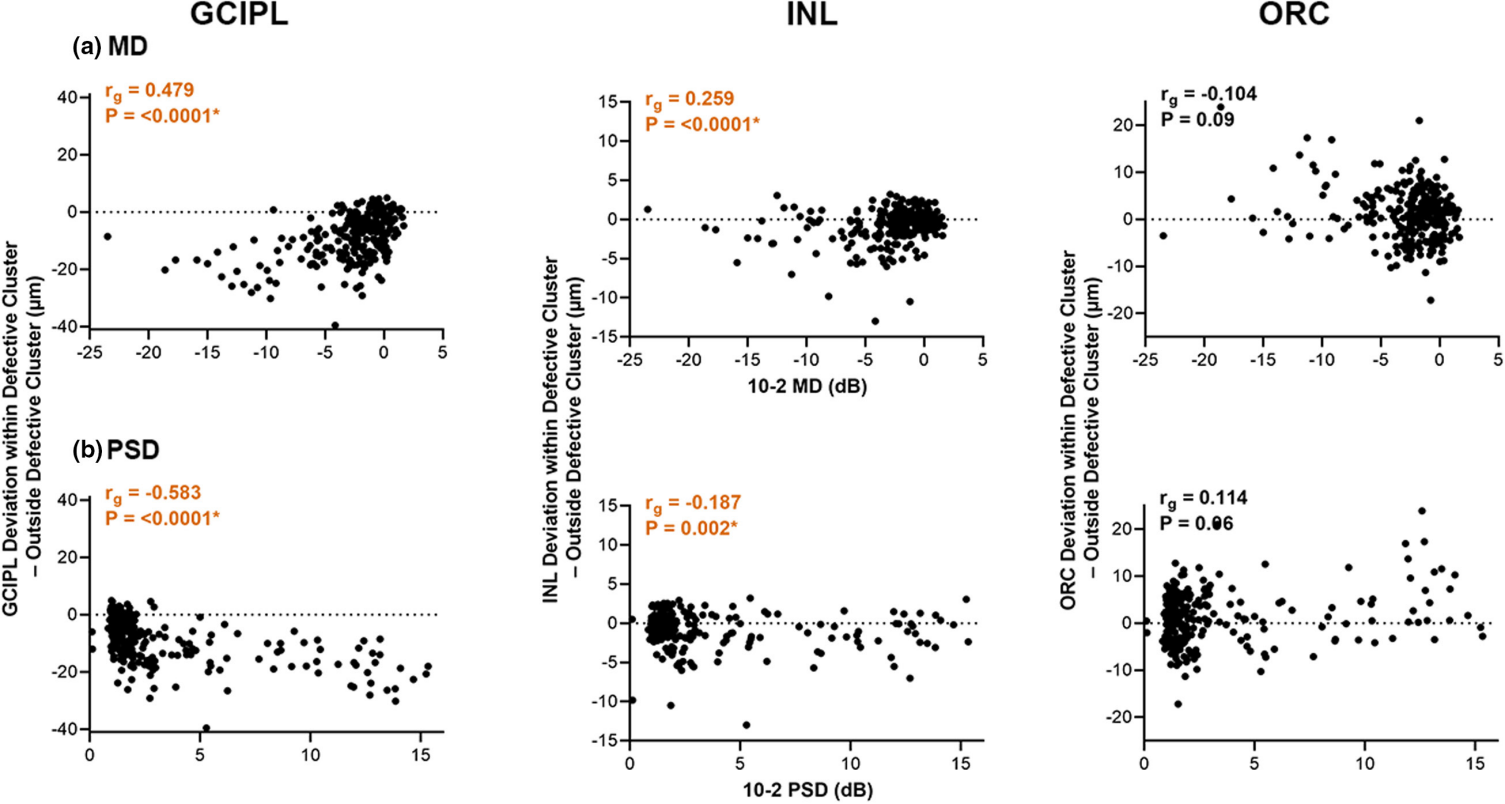
Differences between GCIPL, INL and ORC deviations from the healthy cohort within and outside defective clusters, as per cluster patterns in Figures 2, 3, 4, 5 and Table S1. Mean deviation (MD, a) and pattern standard deviation (PSD, b) were both used as measures of glaucoma severity. Global Spearman's correlations (*r*_*g*_) with glaucoma severity are also shown, with asterisks and coloured text indicate significant correlations. Orange text highlights *r*_*g*_ indicating increasingly negative differences within and outside the most defective clusters observed with increasing glaucoma severity (more negative MD and more positive PSD)

Relationships between retinal thickness and glaucoma participants' VF summary scores were investigated to enable more quantitative comparisons between GCIPL, INL and ORC retinal thickness deviations and observe whether these varied with increasing glaucoma severity. In comparisons with retinal thickness deviations within and outside the most defective GCIPL clusters, negative differences were observed in the GCIPL for 240 glaucoma participants (88.56%), indicating greater reductions within the defective cluster, and moderate correlations with 10‐2 MD and PSD indicated that greater reductions were noted with increasing glaucoma severity (global Spearman's correlation coefficient [*r*_*g*_] 0.479 for MD and −0.583 for PSD, *p* = <0.0001 for both, Figure 6).^63^ Meanwhile, negative differences in INL and ORC deviations within and outside the most defective GCIPL cluster were observed in 154 and 106 glaucoma participants, respectively (56.83% and 39.11%, respectively), indicating greater variability contributing to less consistent trends across all participants. However, weak but significant correlations were observed in INL comparisons, indicating greater reductions in INL thickness with increasing glaucoma severity (*r*_*g*_ = 0.259 for MD and −0.187 for PSD, *p* = <0.0001 for both). By contrast, borderline significant correlations between glaucoma severity and ORC comparisons were observed (*r*_*g*_ = −0.104 for MD, 0.114 for PSD, *p* = 0.09 and 0.06, respectively).

Subsequently, for each glaucoma participant, correlation coefficients were derived across the entire macular region for GCIPL versus INL, GCIPL versus ORC and INL versus ORC, to directly quantify relationships between deviations in retinal layers. For GCIPL‐INL comparisons, 184 participants demonstrated a weak‐to‐moderate positive correlation (67.90%, mean ± SD *r* = 0.282 ± 0.132), indicating that in most glaucoma participants, a decrease in the GCIPL corresponded to a colocalised decrease in INL thickness. Across all participants, overall weak but significant correlations indicated that this positive relationship between GCIPL and INL deviations improves with increasing glaucoma severity (*r*_*g*_ = −0.175 for MD and 0.154 for PSD, *p* = 0.004 and 0.01, respectively). On the contrary, for GCIPL‐ORC comparisons, 79 participants demonstrated a weak‐to‐moderate positive correlation, while 112 demonstrated a weak‐to‐moderate negative correlation (29.15%, mean ± SD *r* = 0.267 ± 0.111 and 41.33%, mean ± SD *r* = −0.249 ± 0.120, respectively), and for INL‐ORC comparisons, 87 and 52 participants demonstrated positive versus negative correlations, respectively (32.10%, mean ± SD *r* = 0.203 ± 0.085 and 19.19%, mean ± SD *r* = −0.194 ± 0.070). These relatively low values highlight that the relationships between the ORC and GCIPL or INL were inconsistent across individuals with glaucoma. Moreover, weak but significant correlations between GCIPL and ORC deviations indicated an increasingly negative relationship with increasing glaucoma severity (*r*_*g*_ = 0.256 for MD, −0.207 for PSD, *p* = <0.0001 for both), indicating that greater reductions in GCIPL thickness were accompanied by apparent increases in ORC thickness at corresponding locations. Similar correlations were observed between INL and ORC deviations, although these were not significant when compared with PSD (*r*_*g*_ = 0.149 for MD and −0.041 for PSD, *p* = 0.01 and 0.50, respectively).

## DISCUSSION

In this proof‐of‐concept study, mutual reductions in macular GCIPL and INL thickness can be observed in glaucomatous eyes, which corresponded in both location per topographic maps generated using cluster analysis and in the magnitude of deviation from matched normative data per individual correlation analyses. However, an absence of change to possible slight thickening in the ORC was observed corresponding to reductions in the GCIPL. Overall, in conjunction with the presiding notion that glaucomatous damage begins at the retinal ganglion cell level, these findings support the theory that trans‐synaptic retrograde degeneration may occur due to glaucoma, with the subtle reduction in INL thickness potentially corresponding to subsequent loss of INL neurons. Alternatively, these findings suggest concurrent loss of retinal ganglion cells and INL neurons, even in relatively early stages of disease.

### Comparison to existing literature

**FIGURE 7:**
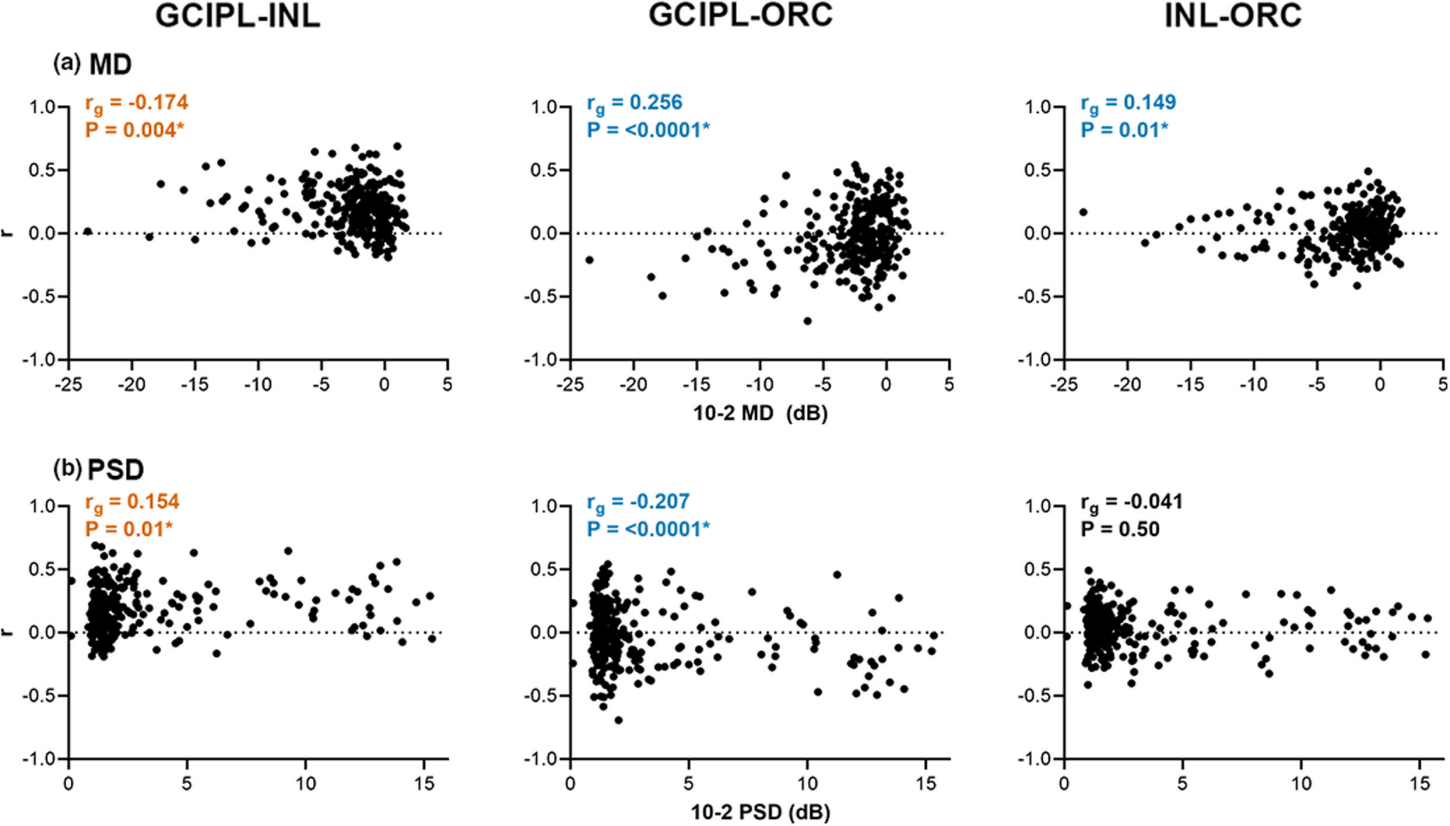
Spearman correlations between glaucoma severity and individual glaucoma participants' Spearman's correlation coefficients (*r*) between GCIPL and INL, GCIPL and ORC, and INL and ORC deviations from the healthy cohort. Mean deviation (MD, a) and pattern standard deviation (PSD, b) were both used as measures of glaucoma severity. Asterisks and coloured text indicate significant correlations. Orange text highlights global correlations (*r*_*g*_) where increasingly positive *r*'s were observed with increasing glaucoma severity (more negative MD and more positive PSD). Conversely, blue text highlights correlations where increasingly negative *r*'s were observed with increasing glaucoma severity.

Previous studies investigating macular retinal layer thickness measurements in glaucoma have variably reported no significant differences in INL and outer retinal thicknesses between glaucoma and comparative cohorts^10^, ^11^, ^12^, ^13^, ^14^, ^15^ and increased INL thickness with increasing glaucoma severity.^13^, ^16^, ^17^, ^18^ Conversely, this study observed colocalised reductions in INL and GCIPL thickness, and two key points have likely contributed to these differences between study findings. First, several studies pooled all glaucoma participants prior to analysis; however, glaucoma can exhibit interindividual variations on its effects on the superior and inferior hemifields of the macula^19^, ^20^; pooling data demonstrating variable locations and extents of macular damage may have masked subtle hemifield‐specific results. Indeed, the heterogeneity in glaucoma was observed in this study's cohort, with notable scatter visible in correlation analyses despite attempts to minimise sources of normal interindividual variability (Figures 6 and 7). Moreover, calculation of average retinal thickness measurements across macular hemifields was performed in studies that did stratify analyses by hemifields corresponding to VF defects,^10^, ^14^, ^17^ so smaller location‐specific changes in the INL and outer retinal layers may have been masked. By minimising factors contributing to normal interindividual variability, including the use of differences in retinal thickness to matched healthy eyes rather than absolute retinal thickness values, the resultant topographic maps generated from cluster analysis revealed locations of INL reduction were typically narrower than colocalised clusters corresponding to GCIPL reduction (Figures 3 and 4). Following on, measurements averaged over standard grids in commercially available software, such as the ETDRS and 8 × 8 grids, demonstrate limited sampling resolution that may have been insufficient to detect the subtle changes in the INL. Differences between customised high‐density grids, akin to that applied in this study, and standard grids have been previously investigated by our group, with high‐density grids enabling identification of eccentricity‐dependent variations in retinal thickness more closely aligning with histology.^26^, ^52^ Overall, this suggests that high sampling resolution and stratification by likely location of glaucomatous defects was beneficial in enabling more accurate pattern visualisation in retinal thickness across the investigated layers.

A potential trend of increasing ORC thickness in regions of GCIPL damage was also observed in this study, although with reasonable variability observed across correlation analyses. This appears inconsistent with previous OCT‐based studies reporting no change or potential slight decrease in outer retinal thickness in glaucoma,^13^, ^16^, ^17^, ^18^ nor with reported decreased cone density, photoreceptor outer segment length, interdigitation zone size and ellipsoid zone reflectivity in locations of inner retinal damage in glaucoma,^64^, ^65^, ^66^ which logically should not translate to an increase in outer retinal thickness. However, reductions in retinal nerve fibre layer reflectivity secondary to glaucomatous damage may affect segmentation of the underlying GCIPL, INL and ORC.^67^, ^68^ Indeed, Darma et al.^69^ observed an increase in outer nuclear layer plus inner segment layer thickness with decreasing overall signal strength in healthy eyes, attributed to poorer contrast between these layers and the adjacent retinal layers affecting boundary segmentation. As such, alterations in signal strength may have disproportionately affected ORC thickness measurements beneath regions of retinal nerve fibre layer damage, potentially contributing to the trends observed in this study. While Darma et al.^69^ did not report notable change in INL thickness with decreasing signal strength, this may be a specific property of the segmentation algorithm applied to their data set. As signal strength has been reported to variably affect retinal thickness measurements obtained from different segmentation software,^70^ it may be prudent to explore the impacts of signal strength on the segmentation software used in this study, OCT Segmentation Version 2.11, to better understand its potential contributions to variations in INL thickness.

Alternatively, the apparent contradiction between reduced INL thickness with increasing glaucoma severity observed in this study and increased INL thickness reported by others may be related to the variable presence of microcystic macular oedema in study cohorts.^13^, ^17^, ^18^ While other studies reporting increased INL thickness did not note microcystic oedema in their cohorts, the visibility of microcystic changes in the INL is highly dependent on sufficient OCT resolution.^16^ Nonetheless, additional variability in INL thickness is introduced with microcystic macular oedema, which has been observed in conjunction with increased, decreased and unchanged INL thickness.^71^ Overall, the apparent colocalised reductions in INL and GCIPL thickness observed in this study may indicate a relatively low proportion of eyes with microcystic macular changes relative to other studies.

### Retinal synaptic pathways and potential changes in glaucoma

Given the links between retinal cell parameters and retinal thickness measurements acquired using OCT,^52^, ^72^ understanding of retinal circuitry via histological studies provides a theoretical basis for the observed interactions between GCIPL and INL thickness measurements. In animal models of glaucoma, reductions in retinal ganglion cell dendrite density have been previously identified,^5^, ^6^ and reductions in IPL thickness in human OCT‐based studies similarly suggest that retinal ganglion cell‐INL connections are disrupted in glaucoma.^73^, ^74^, ^75^ Furthermore, in ischaemia/reperfusion studies of the rat retina, both retinal ganglion cell and bipolar cell loss have been reported,^76^, ^77^ indicating potential concurrent or consecutive processes impact viability of both cell types.

There are a multitude of synaptic targets onto retinal ganglion cells, including cone bipolar cells and various amacrine cells.^78^ Rod bipolar cells synapse onto rod‐specific amacrine cells, including AII amacrine cells, which through conventional glycinergic synapses onto OFF bipolar cells and gap junctions onto ON cone bipolar cells convey the scotopic signal to retinal ganglion cells.^79^, ^80^, ^81^ Indeed, Akopian et al.^82^ reported a significant loss of calretinin‐ and GABA‐immunoreactive amacrine cells in their mouse model of glaucoma, with those coupled to retinal ganglion cells via gap junctions demonstrating poorer survivability. Additionally, there is substantial interaction between the rod and cone pathways at the bipolar cell level, as indicated by the presence of the rod‐cone crossover connectome in mammalian retina,^83^ and is likely to influence the synaptic connections formed with retinal ganglion cells at the IPL level. Indeed, a decreased number of docked vesicles in IPL synapses has been reported in a rat model of glaucoma,^84^ which may reflect a reduction in AII amacrine synapses to retinal ganglion cells. As such, it is perhaps unsurprising that retinal ganglion cell degeneration may impact cell morphology and/or function in a range of inner nuclear layer components.

However, it is important to acknowledge that the resolution afforded by spectral‐domain OCT is insufficient to enable visualisation of individual cell components, and therefore the finding of greater reduction in INL thickness in various VF defect categories is not conclusive of INL cell density loss or the presence of retinal trans‐synaptic degeneration. With the emergence of adaptive optics OCT, in vivo visualisation of cell components would be highly valuable to better understand the time frame of changes in INL cell morphology and/or physiology, if any, that could more robustly support the theory of retinal trans‐synaptic degeneration in glaucoma.

### Advantages of investigating the INL in glaucoma

A key problem affecting detection of structural progression in advanced glaucoma is the OCT measurement floor, where no further change in OCT measurements is observed despite ongoing functional progression.^85^ As the correlation analyses in this study indicated greater reductions in INL thickness and a more positive relationship between GCIPL and INL thickness with increasing glaucoma severity, the suggestion of ongoing reductions in INL thickness in advanced glaucoma could translate to an increased dynamic range upon inclusion of the INL in glaucoma analyses. That is, there is potential for detection of structural progression at later stages of disease than afforded by the macular GCIPL alone.^41^ While the present study did not investigate the measurement floors of combined GCIPL‐INL versus GCIPL alone, future work in this area would be valuable to identify the utility of combined retinal layer complexes in advanced glaucoma; for example, if the measurement floor commences at a more severe MD in the GCIPL + INL compared with the GCIPL alone, this would suggest that the GCIPL + INL is a highly useful structural measure for detecting additional structural progression in advanced glaucoma. Additionally, investigations of the INL alone may be valuable for detection of INL thickening on glaucoma progression analyses, as microcystic macular oedema has been reported to occur more commonly in eyes demonstrating rapid progression.^86^ Future studies investigating whether changes in longitudinal INL thickness, particularly increases in INL thickness corresponding to microcystoid macular oedema, are predictive of glaucomatous progression, would help determine the role of the INL in glaucoma‐based OCT analyses.

## LIMITATIONS

Given the complexity in the clinical diagnosis, diversity in glaucoma presentations and the absence of ‘ground‐truth’ reference standards,^30^ the heterogeneity in glaucoma cohorts could mean that the present findings may not be applicable to other glaucoma populations. Following on from this, the limited refractive error range of the glaucoma participants included in this study, primarily due to the limited pool of healthy eyes with high refractive errors, and therefore the findings of this study may not be generalizable to a cohort with high myopia, for example. Further investigation in other glaucoma cohorts would certainly be valuable to determine repeatability of these findings.

Additionally, due to the various demographic factors requiring adjustment via matching and relatively few older healthy participants, the numbers of participants in the matched healthy subgroups were not consistent across all glaucoma participants, with fewer healthy participants per older glaucoma participant. This has the potential for deviation data to be more greatly affected by outliers in the matched healthy data sets. However, this concern would persist should adjustment via regression models have been applied instead, as fitting of regression models would be based on fewer data points at the extremes of the age range. Nonetheless, more expansive normative database would be worthwhile to address these sample size limitations in future.

While the Hodapp–Parrish–Anderson criteria for defining cluster VF defects are commonly applied in clinical research and practice settings, these criteria were designed from 24‐2 VF test grids,^35^, ^87^ and given the increased sparse sampling density of the 24‐2 test grid application to 10‐2 VF test grids may overclassify defects at central locations; further clarification on the most appropriate definitions to determine cluster VF defects using central test grids may be helpful to aid future research in this area. Furthermore, the OCT Segmentation software used in this study did not allow for correction of segmentation error, resulting in exclusion of data which may not have necessarily been excluded if software allowing for manual correction of segmentation was available. The methods described also do not account for potential variability in retinal thickness deviations due to the temporal raphe; however, previous reports describing variability in temporal raphe position suggest that global correction would be inappropriate,^88^, ^89^, ^90^, ^91^ and the OCT scanning protocol used in this study did not provide sufficient resolution to enable visualisation of individual temporal raphes. Finally, as this study was cross‐sectional in design, the duration of glaucomatous change required prior to observing upstream effects on the INL and ORC could not be determined; longitudinal evaluation of the GCIPL, INL and ORC would be valuable to determine whether concurrent changes in retinal layers occur with progressive glaucoma, which would be a useful comparison with the findings of this study.

## CONCLUSION

This proof‐of‐concept study evaluated the relationships between macular GCIPL, INL and ORC thickness, extracted at a high sampling resolution using custom software, across a spectrum of eyes with glaucoma. Colocalised reductions in INL thickness may be observed in conjunction with reduced GCIPL thickness measurements, and a weak but significant correlation suggests that greater reductions in INL thickness may occur with increasing glaucoma severity. By contrast, no consistent change in the ORC was observed. These findings could support the notion that trans‐synaptic retrograde degeneration may occur in glaucoma, although not extending past the INL. These preliminary findings could indicate the potential usefulness of the INL in evaluating changes observed using OCT in glaucoma, although further investigations are required to verify the role of the INL in greater detail.

## AUTHOR CONTRIBUTIONS

**Janelle Tong:** Conceptualization (equal); data curation (equal); formal analysis (equal); investigation (equal); methodology (equal); software (equal); visualization (equal); writing – original draft (equal); writing – review and editing (equal). **Jack Phu:** Conceptualization (equal); data curation (equal); resources (equal); writing – review and editing (equal). **David Alonso‐Caneiro:** Project administration (equal); resources (equal); software (equal); supervision (equal); writing – review and editing (equal). **Sieu K. Khuu:** Project administration (equal); resources (equal); supervision (equal); writing – review and editing (equal). **Michael Kalloniatis:** Conceptualization (equal); funding acquisition (equal); project administration (equal); resources (equal); supervision (equal); writing – review and editing (equal).

## CONFLICT OF INTEREST

No conflicting relationship exists for any author.

## FUNDING INFORMATION

National Health and Medical Research Council of Australia Ideas Grant [NHMRC 1186915].

## Supporting information


Appendix S1.
