# Supplementary material for: High sampling resolution optical coherence tomography reveals potential concurrent reductions in ganglion cell-inner plexiform and inner nuclear layer thickness but not in outer retinal thickness in glaucoma
Source: Ophthalmic Physiol Opt. 2022 Nov 23;43(1):46–63. doi: 10.1111/opo.13065 (PMC10947055; doi:10.1111/opo.13065)
Supplement: Supplementary file 1 — Appendix S1. [file 44402_2023_4301007_MOESM1_ESM.docx]

**Supplementary Material for: High sampling resolution optical coherence tomography reveals potential concurrent reductions in ganglion cell-inner plexiform and inner nuclear layer thickness but not in outer retinal thickness in glaucoma**

Janelle Tong^1,2^, Jack Phu^1,2,3^, David Alonso-Caneiro^4^, Sieu K. Khuu^2^, Michael Kalloniatis^2,5^

1. Centre for Eye Health, University of New South Wales (UNSW), Sydney, NSW Australia

2. School of Optometry and Vision Science, UNSW, Sydney, NSW Australia

3. Faculty of Medicine, University of Sydney, Sydney, NSW Australia

4. Queensland University of Technology, Contact Lens and Visual Optics Laboratory, Centre for Vision and Eye Research, School of Optometry and Vision Science, QLD, Australia

5. School of Medicine (Optometry), Deakin University, Waurn Ponds, Victoria,

Australia

Supplementary Materials: 5 (3 Figures, 1 Analysis, 1 Table)

Funding information: National Health and Medical Research Council of Australia Ideas Grant

[NHMRC 1186915]

Corresponding author:

Prof Michael Kalloniatis

School of Optometry and Vision Science, UNSW

Sydney 2052, NSW Australia

Email: m.kalloniatis@unsw.edu.au


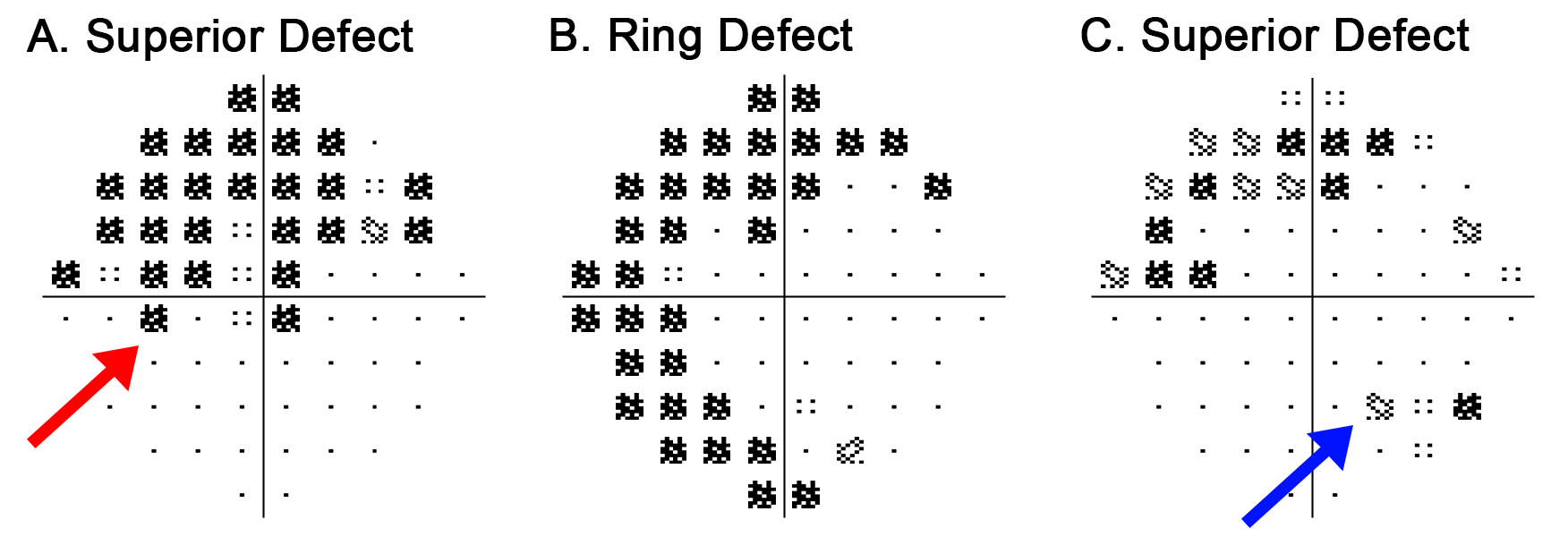


**Supplementary Figure 1.** Examples of visual field (VF) defect types per 10-2 pattern deviation maps, displayed in right eye format. A. A superior defect, defined as a single contiguous VF defective cluster located in the superior hemifield, with at most 1° encroachment on the opposite hemifield (red arrow). B. A ring defect, defined as affecting both hemifields with an arcuate pattern of loss in each hemifield. C. An example with a larger VF defective cluster in the superior hemifield and a smaller cluster in the inferior hemifield (blue arrow). Unlike B., the inferior cluster does not follow an arcuate pattern, and as such classification was based on the hemifield containing the larger cluster, that is the superior hemifield.


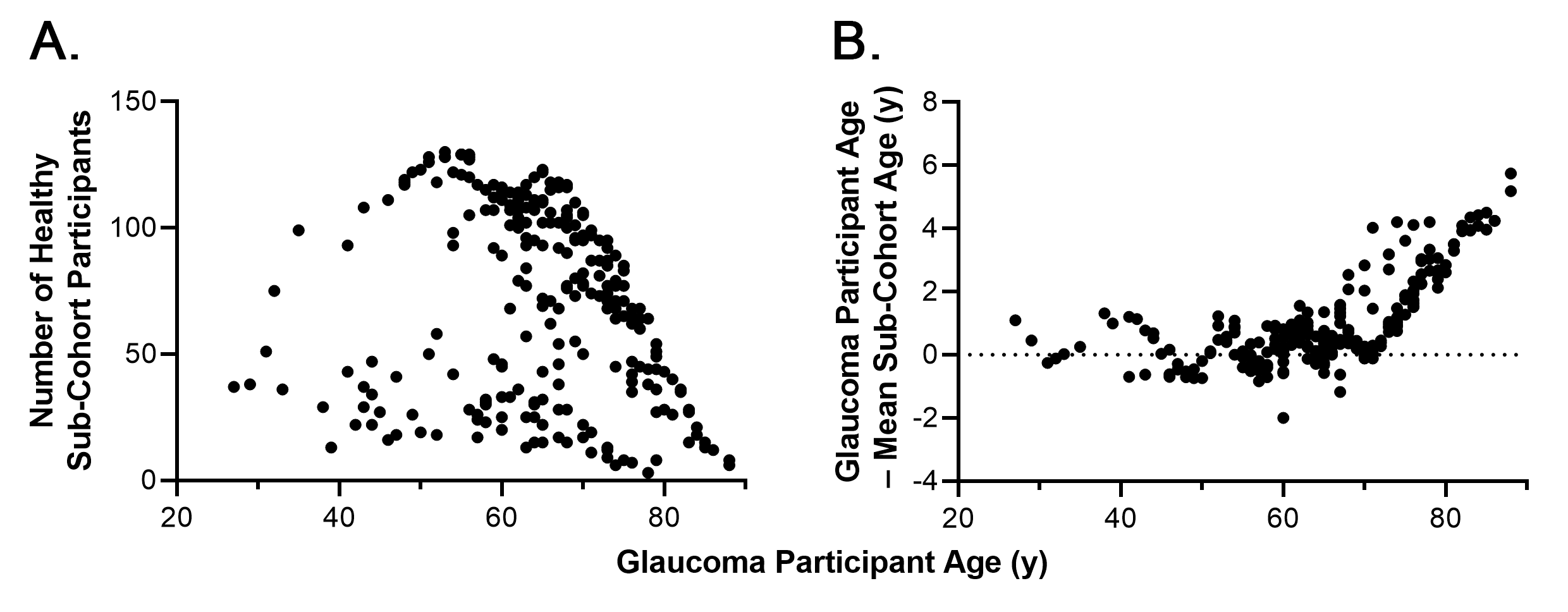


**Supplementary Figure 2.** A. Number of participants in each healthy sub-cohort as a function of glaucoma participant age. B. Differences between glaucoma participant age and mean sub-cohort age as a function of glaucoma participant age.


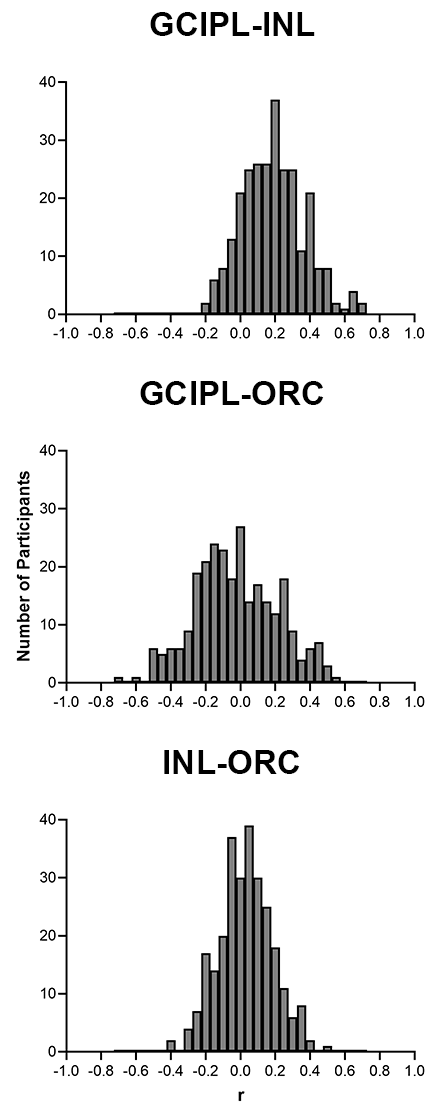


**Supplementary Figure 3.** Frequency histograms of individual glaucoma participants’ Spearman’s correlation coefficients (r) in analyses of the ganglion cell-inner plexiform layer (GCIPL) versus inner nuclear layer (INL), GCIPL versus outer retinal complex (ORC) and INL versus ORC.

**Supplementary Figure 4.** Cluster patterns generated from GCIPL+INL difference maps across the glaucoma cohort, as described in Supplementary Analysis 2, stratified by visual field defect type (columns) and mean deviation criteria (rows). Per Figures 2-5, locations colored the same indicate those that showed no significant difference in retinal thickness deviations from the healthy cohort, matched for age and refractive error per multiple linear regression analyses for GCIPL and INL (Table 3). Mean ± standard deviation retinal thickness deviations in µm are shown for each cluster.


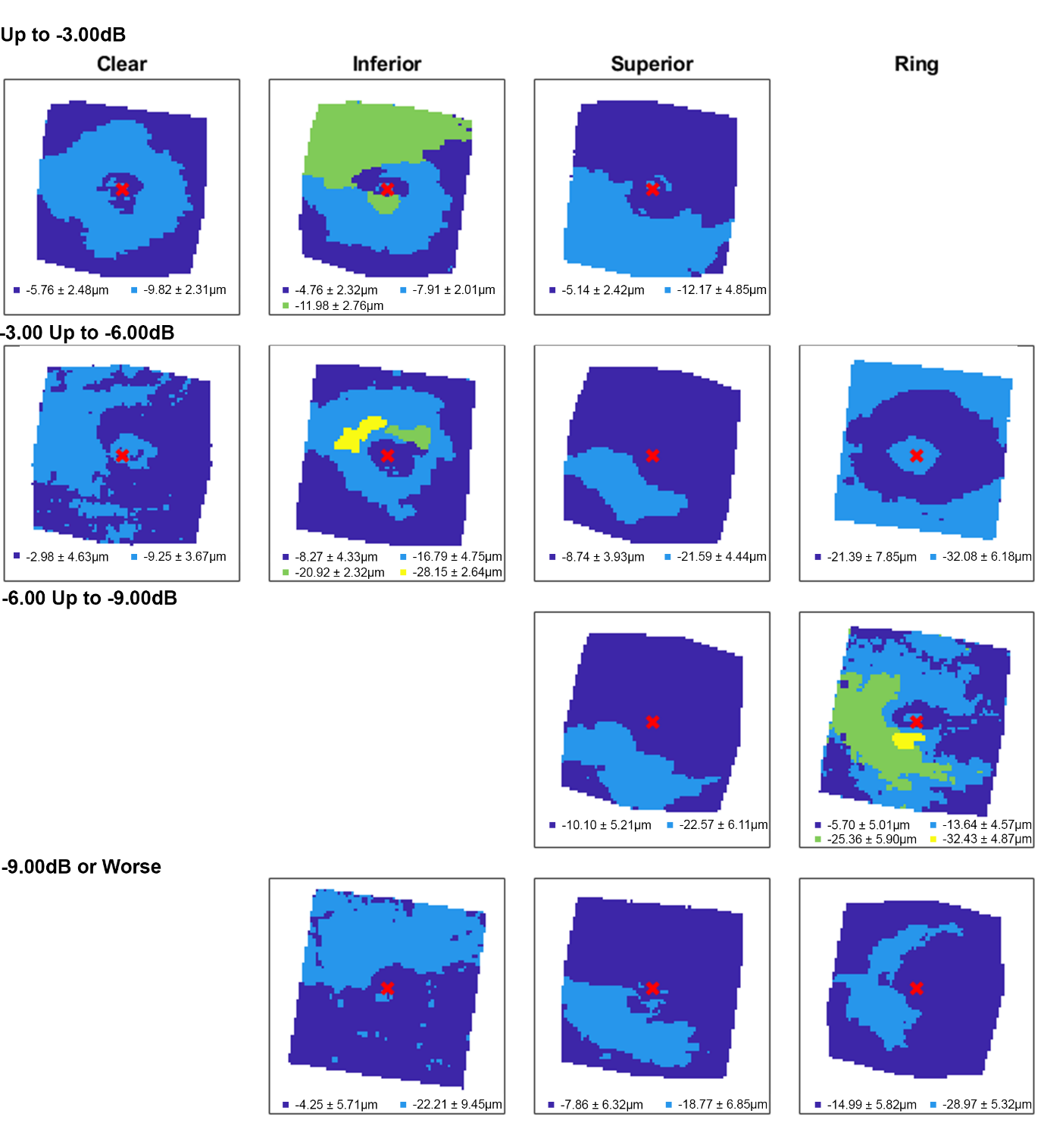


**Supplementary Analysis 1.**

From Supplementary Figure 2B, the maximum difference between glaucoma participant age and mean matched healthy sub-cohort age was 5.75 years, in the oldest glaucoma participant (88 years of age). That is, the mean age of the matched healthy sub-cohort was 82.75 years. To determine investigate whether this maximum difference in ages would produce meaningful differences in retinal thickness, and therefore whether there could be factors other than the presence of glaucoma that could contribute to deviations in retinal thickness, the difference in retinal thicknesses averaged across the entire macula at 82.75 and 88 years of age were calculated from previously described age-regression models.^1^ As these regression models described ageing in the ganglion cell layer (GCL), inner plexiform layer (IPL), outer nuclear layer (ONL) and inner segment-outer segments of the photoreceptors (ISOS), rather than the combined complexes used in the present study, the regression models were used to calculate differences in these individual layers, and the differences in GCL and IPL were summed to determine the difference in the GCIPL, and the difference in ONL and ISOS were summed to determine the difference in the ORC. For average GCIPL, INL and ORC thicknesses across the entire macula, regression models predicted differences of -1.87µm, -0.65µm and -0.68µm respectively, equivalent to percentage differences of -3.13%, -2.26% and -0.54% of 88 year old data. These small differences in retinal thickness for the scenario of maximum age difference imply that age-related decline in retinal thickness would minimally contribute to the differences between glaucoma and healthy sub-cohort data, and that the chosen age range for matching of ±7.5 years is appropriate.

**Supplementary Analysis Reference**

1. Trinh M, Khou V, Zangerl B, Kalloniatis M, Nivison-Smith L. Modelling normal age-related changes in individual retinal layers using location-specific OCT analysis. *Sci Rep* 2021;11(1):558.

**Supplementary Analysis 2.** As consistent co-localized changes in retinal thickness were observed between the GCIPL and INL, particularly in eyes with inferior and superior VF defects, these layers were combined and re-analyzed per methods applied to individual layers, to enable visualization of patterns of change across the glaucoma spectrum. That is, after deviation data from the glaucoma cohort relative to matched healthy eye data were calculated, hierarchical cluster algorithms were applied to data stratified per glaucoma subcohort, with the final number of clusters determined using Bayesian Information and above d’ separation criteria. Generally, cluster patterns generated for the GCIPL+INL were qualitatively similar to those generated for the GCIPL, other than participants with ring VF defects and MD up to -6.00dB demonstrating paracentral ring-shaped regions of slightly thicker GCIPL+INL. Overall, this suggests that deviations in GCIPL+INL thickness adequately reflect alterations in GCIPL thickness in this glaucoma cohort.

**Supplementary Table 1.** Mean and standard deviation retinal thicknesses in the glaucoma cohort from the healthy cohort, matched per demographic characteristics, pooled across the entire macula and each cluster as shown in Figures 2-5 and Figure 8. For all, the lowest numbered cluster indicates locations demonstrating the most positive deviation from the healthy cohort, that is either an increase or smallest reduction in retinal thickness, and the highest numbered cluster indicates location demonstrating the largest negative deviation or reduction in retinal thickness. All measurements are in microns (µm).

| **GCIPL** | **Average** | **Cluster 1** | **Cluster 2** | **Cluster 3** | **Cluster 4** |
| --- | --- | --- | --- | --- | --- |
| **Up to -3.00, C** | -6.79 ± 2.76 | -4.94 ± 2.00 | -8.86 ± 1.87 |  |  |
| **Up to -3.00, I** | -7.52 ± 3.46 | -4.70 ± 2.07 | -7.28 ± 2.01 | -11.62 ± 2.33 |  |
| **Up to -3.00, S** | -6.84 ± 4.45 | -4.50 ± 2.11 | -9.19 ± 3.57 | -18.91 ± 2.05 |  |
| **-3.00 up to -6.00, C** | -4.86 ± 4.29 | -2.70 ± 3.70 | -6.65 ± 3.90 |  |  |
| **-3.00 up to -6.00, I** | -9.58 ± 5.64 | -6.21 ± 3.55 | -12.57 ± 3.27 | -20.83 ± 3.35 |  |
| **-3.00 up to -6.00, S** | -8.92 ± 5.33 | -7.06 ± 3.08 | -17.95 ± 4.64 |  |  |
| **-3.00 up to -6.00, R** | -8.26 ± 6.23 | -6.41 ± 3.79 | -21.19 ± 4.29 |  |  |
| **-6.00 up to -9.00, S** | -10.07 ± 6.06 | -8.20 ± 4.06 | -19.91 ± 5.22 |  |  |
| **-6.00 up to -9.00, R** | -10.94 ± 7.76 | -6.22 ± 5.24 | -12.03 ± 4.78 | -22.06 ± 4.65 |  |
| **-9.00 or worse, I** | -8.00 ± 8.66 | -2.10 ± 4.11 | -10.62 ± 2.21 | -16.27 ± 5.17 | -34.38 ± 3.45 |
| **-9.00 or worse, S** | -9.37 ± 7.00 | -5.67 ± 4.84 | -11.43 ± 2.86 | -15.79 ± 4.82 | -29.28 ± 4.08 |
| **-9.00 or worse, R** | -13.77 ± 6.74 | -11.31 ± 4.60 | -17.23 ± 3.97 | -25.62 ± 5.67 |  |
| **INL** | **Average** | **Cluster 1** | **Cluster 2** | **Cluster 3** |  |
| **Up to -3.00, C** | -0.92 ± 0.69 | 1.99 ± 0.19 | -0.55 ± 0.58 | -1.24 ± 0.59 |  |
| **Up to -3.00, I** | -0.87 ± 0.95 | -0.54 ± 0.68 | -2.13 ± 0.79 |  |  |
| **Up to -3.00, S** | -1.23 ± 1.18 | -0.83 ± 0.75 | -3.06 ± 1.04 |  |  |
| **-3.00 up to -6.00, C** | -0.56 ± 1.96 | -0.43 ± 1.85 | -4.02 ± 1.51 |  |  |
| **-3.00 up to -6.00, I** | -2.51 ± 1.68 | -2.27 ± 1.42 | -5.78 ± 1.63 |  |  |
| **-3.00 up to -6.00, S** | -2.03 ± 1.67 | -1.68 ± 1.48 | -4.02 ± 1.29 |  |  |
| **-3.00 up to -6.00, R** | -2.80 ± 2.25 | -2.32 ± 1.78 | -6.99 ± 1.46 |  |  |
| **-6.00 up to -9.00, S** | -2.97 ± 2.38 | -2.68 ± 2.19 | -6.44 ± 1.77 |  |  |
| **-6.00 up to -9.00, R** | -2.49 ± 2.82 | -1.25 ± 1.93 | -3.99 ± 2.24 | -6.78 ± 2.50 |  |
| **-9.00 or worse, I** | -3.60 ± 4.09 | -1.46 ± 2.62 | -6.79 ± 3.81 |  |  |
| **-9.00 or worse, S** | -1.05 ± 1.92 | -0.52 ± 1.62 | -3.19 ± 1.49 |  |  |
| **-9.00 or worse, R** | -3.95 ± 2.78 | -2.68 ± 2.06 | -6.62 ± 2.11 |  |  |
| **ORC** | **Average** | **Cluster 1** | **Cluster 2** | **Cluster 3** |  |
| **Up to -3.00, C** | -0.02 ± 1.69 | 2.60 ± 0.51 | -0.04 ± 1.68 |  |  |
| **Up to -3.00, I** | 0.10 ± 1.61 | 2.37 ± 1.52 | 0.94 ± 1.16 | -0.37 ± 1.39 |  |
| **Up to -3.00, S** | -1.46 ± 1.95 | -1.23 ± 1.34 | -9.68 ± 2.68 |  |  |
| **-3.00 up to -6.00, C** | 1.04 ± 4.13 | 10.21 ± 2.34 | 0.66 ± 3.74 |  |  |
| **-3.00 up to -6.00, I** | -2.57 ± 2.84 | -2.46 ± 2.81 | -5.57 ± 1.84 |  |  |
| **-3.00 up to -6.00, S** | -1.53 ± 2.12 | 0.07 ± 1.37 | -1.75 ± 2.00 |  |  |
| **-3.00 up to -6.00, R** | -6.08 ± 5.90 | -5.88 ± 3.70 | -20.81 ± 3.09 |  |  |
| **-6.00 up to -9.00, S** | -4.03 ± 2.69 | -2.18 ± 2.00 | -5.03 ± 2.21 | -8.17 ± 1.28 |  |
| **-6.00 up to -9.00, R** | -1.48 ± 4.11 | 2.86 ± 3.97 | -1.84 ± 3.42 |  |  |
| **-9.00 or worse, I** | -1.86 ± 6.03 | 2.96 ± 3.69 | -2.83 ± 4.12 | -21.04 ± 3.83 |  |
| **-9.00 or worse, S** | 2.56 ± 3.77 | 3.01 ± 2.94 | -9.58 ± 3.41 |  |  |
| **-9.00 or worse, R** | -0.44 ± 3.57 | 4.02 ± 1.90 | -0.84 ± 3.41 |  |  |
| **GCIPL+INL** | **Average** | **Cluster 1** | **Cluster 2** | **Cluster 3** | **Cluster 4** |
| **Up to -3.00, C** | -7.70 ± 3.14 | -5.76 ± 2.48 | -9.82 ± 2.31 |  |  |
| **Up to -3.00, I** | -8.39 ± 3.86 | -4.76 ± 2.32 | -7.91 ± 2.01 | -11.98 ± 2.76 |  |
| **Up to -3.00, S** | -8.07 ± 5.03 | -5.14 ± 2.42 | -12.17 ± 4.85 |  |  |
| **-3.00 up to -6.00, C** | -5.41 ± 5.26 | -2.98 ± 4.63 | -9.25 ± 3.67 |  |  |
| **-3.00 up to -6.00, I** | -12.09 ± 6.73 | -8.27 ± 4.33 | -16.79 ± 4.75 | -20.92 ± 2.32 | -28.15 ± 2.64 |
| **-3.00 up to -6.00, S** | -10.95 ± 6.3 | -8.74 ± 3.93 | -21.59 ± 4.44 |  |  |
| **-3.00 up to -6.00, R** | -27.01 ± 8.82 | -21.39 ± 7.85 | -32.08 ± 6.18 |  |  |
| **-6.00 up to -9.00, S** | -13.04 ± 7.59 | -10.10 ± 5.21 | -22.57 ± 6.11 |  |  |
| **-6.00 up to -9.00, R** | -13.43 ± 9.13 | -5.70 ± 5.01 | -13.64 ± 4.57 | -25.36 ± 5.90 | -32.43 ± 4.87 |
| **-9.00 or worse, I** | -11.6 ± 11.56 | -4.25 ± 5.71 | -22.21 ± 9.45 |  |  |
| **-9.00 or worse, S** | -10.43 ± 7.94 | -7.86 ± 6.32 | -18.77 ± 6.85 |  |  |
| **-9.00 or worse, R** | -17.72 ± 7.97 | -14.99 ± 5.82 | -28.97 ± 5.32 |  |  |

*C, clear; I, inferior; S, superior; R, ring.*
